# Determining the impact of age and sex on the psychophysical and neurophysiological response to thermal pain across the adult lifespan

**DOI:** 10.1111/jan.14514

**Published:** 2021-01-15

**Authors:** Sebastian W. Atalla, Ronald L. Cowan, Alison R. Anderson, Mary S. Dietrich, Larkin Iversen, Laura Beth Kalvas, Karen O. Moss, Kathy Wright, Todd B. Monroe

**Affiliations:** ^1^ The Ohio State University College of Nursing Columbus OH USA; ^2^ Vanderbilt University Medical Center Psychiatric Neuroimaging Program Nashville TN USA; ^3^ Vanderbilt University Medical Center Institute of Imaging Science Nashville TN USA; ^4^ Vanderbilt University Department of Psychiatry and Behavioral Sciences Nashville TN USA; ^5^ Vanderbilt University School of Nursing Nashville TN USA

**Keywords:** adult lifespan, experimental pain, functional MRI, nursing, older adults, research protocol, sex‐differences

## Abstract

**Aims:**

Determine sex‐ and age‐associated psychophysical and neurophysiological differences in the processing of pain across the adult lifespan.

**Design:**

Preliminary, exploratory, cross‐sectional study.

**Methods:**

Using psychophysics (to measure intensity and unpleasantness) and functional magnetic resonance imaging blood oxygenation level dependent methods (to measure stimulus‐evoked brain activation), we will examine sex‐ and age‐associated differences in thermal pain processing and their underlying neurophysiology in a broad range of healthy adults (ages 30–89). We will acquire resting state functional connectivity data for secondary analyses exploring whether resting state connectivity predicts psychophysical and neurophysiological responses to thermal pain. To examine the effects of altered blood flow, we will acquire resting‐state arterial spin labeling magnetic resonance imaging data to quantify resting cerebral blood flow. We will interpret findings in the context of a proposed neural model of pain, ageing, and sex. Study funding was received in June of 2014. Ethical approval was obtained from the Vanderbilt University IRB prior to study initiation.

**Conclusion:**

Exploring the biological reasons for age‐ and sex‐associated differences in pain processing will increase our understanding of pain in older adults. The paucity of neurobiological evidence to support best practice pain management in older adults places these individuals at risk for poor pain management.

**Impact:**

Poorly treated pain in older adults is a critical public health problem associated with a poor quality of life and increased healthcare costs. Understanding how age and sex have an impact on central processing of pain across the lifespan is a critical step toward improving personalized pain medicine.

## 摘要

**目的:**

确定成年期疼痛处理中与性别和年龄相关的心理、生理和神经生理学差异。

**设计:**

初步、探索性、分类排列研究。

**方法:**

利用心理物理学 (测量强度和不愉快程度) 和功能磁共振成像血氧水平依赖法 (测量刺激诱发的脑激活) , 我们将在广泛的健康成年人 (30‐89岁) 中研究与性别和年龄相关的热痛处理差异及其潜在的神经生理学。我们将获得静息状态功能连接数据, 用于二次分析, 以便探索静息状态连接是否能预测热痛的心理、生理和神经生理学反应。为检测血液流动改变的影响, 我们将获取静息状态动脉自旋标记磁共振成像数据, 以量化静息脑血流。我们将按照提出的疼痛、衰老和性别的神经模型解释这些发现。我们已于2014年6月收到研究资金。研究开始前, 我们已获得范德堡大学IRB的伦理批准。

**结论:**

探索与年龄和性别相关的疼痛处理差异的生物学原因将增加我们对老年人疼痛的了解。缺乏神经生物学证据来支持老年人疼痛管理的最佳实践, 将这些个体置于疼痛管理不善的风险之中。

**影响:**

老年人疼痛治疗不当是严重的公共卫生问题, 其与生活质量差和保健费用增加有关。了解年龄和性别对整个生命周期内疼痛中枢处理的影响是改善个体化疼痛医学的关键一步。

## 1 INTRODUCTION

Poorly treated pain in older adults is a critical public health problem. Seventy percent of older adults report some level of pain (Thomas, Peat, Harris, Wilkie, & Croft, 2004), with the numbers expected to increase with the ageing of the world's population. Compared with young adults, older adults have more painful diagnoses, increased pain thresholds and are at risk for sub‐optimal treatment of their pain (Hadjistavropoulos & Fine, 2006). Sex‐associated differences in pain are reported in the literature, with women generally experiencing more pain and reporting increased pain sensitivity (Berkley, 1997; Institute of Medicine (US) Committee on Understanding the Biology of Sex and Gender Differences, 2001). Poorly treated pain leads to many associated symptoms, has a negative impact on quality of life and increases healthcare costs (AGS Panel on Persistent Pain in Older Persons, 2002; Bryant, Grigsby, Swenson, Scarbro, & Baxter, 2007; Leveille, Fried, & Guralnik, 2002; Thomas et al., 2004). Understanding age‐ and sex‐associated differences in pain processing between younger and older adults will provide mechanistic insight into factors that underlie the altered pain of the older adult population and provide a critical step to improving personalized pain medicine (Helme & Gibson, 2001).

### 1.1 Background

Pain is the number one reason that people seek medical attention (The American Pain Foundation, 2011). The prevalence of painful conditions increases with age (Hadjistavropoulos & Fine, 2006); 70% of older adults report some level of pain and 38% have pain that interferes with activities of daily living (Thomas et al., 2004). With the ageing of the world's population, these numbers are expected to increase. Relative to males, females experience considerably more chronic pain and are exposed more frequently to painful conditions (Berkley, 1997; Institute of Medicine (US) Committee on Understanding the Biology of Sex and Gender Differences, 2001). Understanding sex differences in pain processing between younger and older adults will provide mechanistic insight into factors that underlie the altered pain experience and increased chronic pain risk in the older adult population (Helme & Gibson, 2001).

**FIGURE 1:**
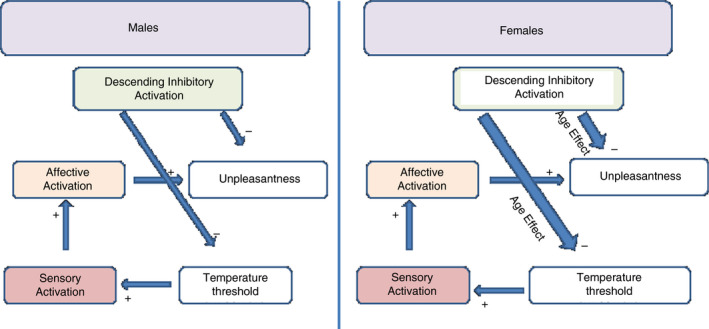
Proposed neural model of pain, ageing, and sex

Ageing is associated with changes in peripheral nociceptive fiber function and with structural and functional brain alterations in pain regions. These include a reduction in the ratio of fast conducting Aδ to slower conducting C‐fibers and an overall reduction in sensory fiber density (Caterina, Gold, & Meyer, 2005, 2009). This fiber shift may have important implications for interpreting subjective pain reporting in older adults because C‐fiber transmission is associated with the affective pain response and chronic pain. A single report in a very small sample found reduced activation to thermal pain in some brain regions in older adults, yet the association of sex with activation was not reported (Quiton et al., 2007). Based on the existing literature and our preliminary findings, we developed a causative model linking age and sex effects on pain processing (Figure 1). We predict age‐associated increases in thermal thresholds and age‐associated decreases in unpleasantness, along with age‐associated increases in activation of sensory and affective brain regions, during experimental thermal pain. Furthermore, we predict that these changes are more pronounced in females, with females showing an overall greater activation in brain regions mediating descending inhibition of pain.

The experience of pain is associated with structural and functional brain changes. During ageing, cumulative lifetime experience of pain increases, with females having greater lifetime exposure to pain compared with males. Ageing is associated with gray matter volume changes (Smith, Chebrolu, Wekstein, Schmitt, & Markesbery, 2007), including loss of brain volume in pain processing regions (Raz et al., 2005; Walhovd et al., 2011). Older adults are at risk of poor pain management but the neurobiological basis for pain under treatment in older adults remains unknown. Functional magnetic resonance imaging (fMRI) measures of brain activation in specific brain regions show neural signatures representing the experience of pain.(Wager et al., 2013) As such, understanding sex and age effects on psychophysics and brain activation during thermal pain processing provides a foundation for a range of clinically relevant pain assessments and interventions.

The density of epidermal nerve fibers has been shown to decrease with age and is lower in men compared with women (Gøransson, Mellgren, Lindal, & Omdal, 2004). Some researchers have found that women and men report similar pain intensity for lower temperatures, while others have found that women report lower pain thresholds for lower temperatures (Bartley & Fillingim, 2013; Cole, Farrell, Gibson, & Egan, 2010). Neuroimaging studies demonstrate sex effects on brain activation during pain processing in younger cohorts (Labus et al., 2008). Depression is associated with greater pain and more unpleasantness in women, while anxiety is associated with greater pain sensitivity in men (Riley, Robinson, Wade, Myers, & Price, 2001).

In summary, ageing‐related peripheral and central nervous system changes may lead to altered pain processing in older adults. The mechanisms linking psychophysical and central neurophysiological responses to cutaneous thermal pain in ageing are poorly understood. This protocol is designed to address the following critical unanswered questions: (a) How do thermal pain sensory and affective reports change with ageing and do these differ by sex? (b) How do psychophysical sensory and affective measures map onto brain activation during cutaneous thermal stimulation ranging from just noticeable pain to moderate pain? (c) Which brain regions are most strongly implicated in age and sex‐associated changes in brain responses to thermal pain stimulation? (d) Do age‐associated shifts in the function of brain regions mediating descending inhibition of pain differ by sex and does activation in these regions predict subjective reporting of pain?

## 2 THE STUDY

### 2.1 Aims

The goals of this proposed study are to examine age and sex effects on psychophysical measures (sensory threshold and affective/unpleasantness) and neurophysiological measures (stimulus evoked activation) in response to experimental cutaneous thermal pain delivery and to determine how psychophysical and neurophysiological measures of pain are related.

#### 2.1.1 Aim 1

Determine whether sensory (stimulus intensity perceptual threshold) and affective (stimulus rated self‐report of unpleasantness) responses are associated with ageing and sex during cutaneous thermal stimulation using psychophysical parameters.

Hypothesis 1 Age will be positively associated with sensory thresholds (in degrees Celsius) in females but less so in males during the perception of pain. Females will report lower thresholds for pain detection than age‐matched males.

Hypothesis 2 Age will be negatively associated with unpleasantness ratings during pain detection in females, but less so in males. Females will report less unpleasantness than age‐matched males.

#### 2.1.2 Aim 2

Determine whether brain activation is associated with ageing and sex during the application of cutaneous thermal stimulation using neurophysiological parameters.

Hypothesis 1 Age will be positively associated with brain activation in the sensory pain regions in females but less so in males. However, females will demonstrate greater brain activation in sensory pain regions relative to males.

Hypothesis 2 Age will be positively associated with brain activation in the affective pain regions in females but less so in males during the delivery of experimental thermal pain. When compared with males, females will demonstrate greater brain activation in regions mediating descending inhibition of pain.

### 2.2 Design

Using psychophysics to measure thermal sensory threshold and affective unpleasantness and the fMRI blood oxygenation level dependent (BOLD) method (Ogawa, Lee, Nayak, & Glynn, 1990) to measure stimulus‐evoked brain activation, we will examine age‐associated differences in thermal pain processing across much of the adult lifespan (age 30–89). We will also explore the relationship between resting‐state functional connectivity and pain responses to determine if resting‐state connectivity predicts psychophysical and neurophysiological responses to thermal pain. Functional neuroimaging methods incorporating BOLD signal are the gold‐standard for measuring changes in brain function via changes in blood oxygenation; when coupled with an in‐scanner task, changes in the BOLD signal throughout the duration of the task paradigm may be assessed in conjunction with other confounds and covariates to garner a more detailed understanding of neurological processes that are engaged during a task or during rest (Gauthier & Fan, 2019).

To control for the effects of altered blood flow associated with ageing (Melamed, Lavy, Bentin, Cooper, & Rinot, 1980), we will acquire resting‐state arterial spin labeling (ASL), a non‐invasive and non‐ionizing way to trace blood flow (Petcharunpaisan, 2010; Petersen, Zimine, Ho, & Golay, 2006), to quantify resting‐state cerebral blood flow (CBF). Quantifying changes in cerebral blood flow will allow us to measure how sex‐differences in CBF across the lifespan may affect pain sensation and perceived pain unpleasantness and afford insights into how treatment and therapy for alterations in pain perception may account for these physiological differences.

We will interpret findings in the context of a proposed neural model of pain, ageing and sex (Figure 1) which predicts age‐associated changes (greater in females than males) in thermal thresholds, unpleasantness and activation of sensory and affective brain regions during experimental thermal pain.

### 2.3 Sample/participants

Subjects will be recruited using emails, letters and flyers circulated in a southeastern university, its academic medical center and the surrounding metropolitan area. Email communications will be managed by institutional mass‐email distribution services and will prioritize prospective participants who have expressed an interest in participating to either a participating physician or a recruitment platform such as ResearchMatch, a national health volunteer registry that was created by several academic institutions and supported by the U.S. National Institutes of Health as part of the Clinical Translational Science Award (CTSA) program. Recruitment letters will be mailed to prospective participants who have either expressed interest in the study to their physician or were identified as candidates through chart review. Flyers will be posted throughout the medical center and will adhere to IRB guidelines outlining transparency and undue coercion; furthermore, these flyers will also be posted throughout the surrounding area at community centers and assisted living facilities. Because we are interested in the continuous measure of age, we will enroll 60 subjects to yield a final continuous sex‐balanced stratified sample of adults aged 30–89. We will stratify by age range (to allot 5 males/5 females) to each of six age strata: 30–39, 40–49, 50–59, 60–69, 70–79, 80–89. We will identify females who are pre‐ and post‐menopausal to control for hormone status on psychophysical and neurophysiological pain.

#### 2.3.1 Inclusion criteria

Participants will be considered for inclusion if they meet the following criteria: (a) ages 30–89; (b) right‐handed; (c) English speaking; (d) verbally communicative and able to provide a pain rating; (e) not taking an analgesic (opioid or non‐narcotic) medication within 1 week of testing; (f) sex‐ and race/ethnicity‐matched; (g) matched on socioeconomic status with consideration for retirement status (Hollingshead, 1975); (h) able to see and hear; (i) education matched; (j) at least one wholly intact ovary; and (k) for pre‐menopausal females, all testing and scanning procedures will occur 2 weeks after the last menstrual period. This process will be verbally verified by the participant during enrollment and MRI procedures scheduled at that time.

#### 2.3.2 Exclusion criteria

Exclusion criterions are first determined through self‐report of health questions and current medications. If questions remain, all subjects will be asked to provide a release of medical records to definitively determine if exclusion criteria are present, including: (a) peripheral neuropathy, diabetes, or stroke; (b) unstable cardiovascular disorder; (c) current or historical alcohol or substance abuse or dependence; (d) Axis I psychiatric disorders; (e) movement disorder (Parkinson's disease or restless leg syndrome); (f) conditions contraindicated for MRI (claustrophobia, pacemaker, ventricular shunt, inability to determine if any metal in the body is 3T safe, unable to obtain medical records for surgical safety clearance); (g) cognitive impairment (Mini Mental State Examination score <27; (Folstein, Folstein, & McHugh, 1975)); (h) severe spinal curvature, spinal disorder, or severe arthritis; (i) acute or chronic pain condition requiring scheduled analgesics; (j) current smoker or previous smoker within the last 5 years; (k) uncontrolled hypertension or hypertension treated with vasoactive medications (e.g. calcium channel blockers; beta blockers; ACE inhibitors, etc.; stable use of diuretics will be permitted); (l) Raynaud's disease; (m) current cancer diagnosis or painful tumor; (n) migraine history; and (o) other reasons as determined by research team. This detailed strategy will result in a particularly healthy set of subjects to help avoid confounds related to co‐morbidities, conditions and medication.

### 2.4 Data collection

The study will take place on 2 days: (a) a screening/enrollment day consisting of consent, study assessments and psychophysical testing and (b) a testing day consisting of the same study assessments and psychophysical testing, followed by neuroimaging acquisition.

#### 2.4.1 Assessments

The Brief Pain Inventory Short Form (BPI‐SF), particularly questions 5 and 6, will be used to collect current and prior pain scores, rated between 0–10 (Mendoza, Mayne, Rublee, & Cleeland, 2006). The BPI‐SF demonstrates, in general, a Cronbach's α between 0.77–0.91. In geriatric populations, the BPI‐SF exhibits a Cronbach's α of at least 0.80 (Kapstad, Rokne, & Stavem, 2010; MD Anderson, n.d). World Health Organization 5‐item well‐being index (WHO‐5) will be used to assess depression. Using DSM‐IV criteria for depression as the index of validity, the WHO‐5 exhibits a Cronbach's α of 0.91 (Löwe et al., 2004). The State Trait (STAI) Anxiety Inventory (Spielberger, 1983) will be used assess anxiety and exhibits a Cronbach's α of at least 0.89 (Grös, Antony, Simms, & McCabe, 2007). While we are excluding individuals with psychiatric diagnoses, lower levels of depression or anxiety may influence outcome measures.

#### 2.4.2 Psychophysical testing (Aim 1)

Using the Medoc PATHWAYS Pain and Sensory Evaluation System (Medoc Ltd., Israel), a thermal stimulus will be delivered in degrees Celsius (°C; starting at 30°C and ramping upward at a rate of 1°C/s) to the thenar eminence of the right hand. We will deliver the thermal stimuli necessary to produce a subjective report of just noticeable pain (JNP), weak pain (WP) and moderate pain (MP). Three trials of each of the three levels of stimuli will be obtained and a single average score for JNP, WP and MP will be recorded for each subject. Next the average temperature will be administered and participants will be immediately be asked to provide an unpleasantness (affective) rating for each percept ranging from 0–20 where 0 indicates neutral and 20 indicates severely distressing for JNP, WP and MP (Petzke, Harris, Williams, Clauw, & Gracely, 2005).

#### 2.4.3 Neurophysiological testing (Aim 2)

After establishing psychophysical parameters for the thermal pain stimulation in Aim 1, subjects will have a brief break and then begin the MRI procedures for Aim 2 to determine whether brain activation is associated with ageing and sex during cutaneous thermal stimulation. The MRI scanner run order will be: (a) structural brain imaging; (b) resting‐state ASL; (c) resting‐state fMRI; and (d) fMRI during cutaneous thermal pain stimulation (4 runs). Total scanner time is 50 min.

Structural brain imaging will be performed with a Philips 3 T Intera Achieva MRI scanner (Philips Medical Systems Andover, MA, USA). A standard whole‐brain 3‐D anatomical T1‐weighted/TFE (with SENSE coil) scan will be acquired for alignment and for segmentation to control for volumetric differences (Karageorgiou et al., 2009). Structural brain changes may account in part for changes in pain function but may also confound interpretation of neuroimaging data. We will therefore enroll subjects without self‐reported chronic pain, and we will control for brain volume components in our imaging analyses. Gray matter atrophy is common in AD brains. The confound of atrophy may result in poorly registered functional activation maps due to significant between‐participants differences in brain structure. Atrophy will be controlled by calculating the standardized residual of gray matter volume relative to total intracranial volume.

Subjects will next undergo thermal pain stimulation during fMRI. The PATHWAYS thermode will be attached to the thenar eminence of the right hand. Stimuli will be based on the average temperature rated as producing JNP, WP, and MP during psychophysics collected in Aim 1. We will use a standard block design (because our primary goal is to detect activation differences) with six thermal stimulation periods (duration 16 s). Each condition is delivered twice per functional run. Baseline is no stimulus (duration 24 s). There are four runs; stimulus order is pseudorandomized for each run and for each subject to avoid order effects. Image acquisition during each 264 s functional run will consist of 28 field echo EPI (132 volumes, 4.40 mm slice thickness with 0.45 mm gap, 2 s TR, 35 ms TE, 79° flip angle, FOV = 240, matrix = 128 × 128) scans.

Next, functional images will be aligned to each other within runs using rigid‐body coregistration in SPM 12 (Penny, Friston, Ashburner, Kiebel, & Nichols, 2007). Images will be coregistered to the T1‐weighted structural image and the transformation of the structural image to MNI space will be applied to the functional images, followed by resampling, yielding functional images at 3mm isotropic resolution. We will spatially smooth with a 6mm‐FWHM Gaussian kernel.

We will use region of interest (ROI) masks from the WFU PickAtlas tool box to delineate the anatomical boundaries for ROIs in our a priori hypotheses (Maldjian, Laurienti, & Burdette, 2004; Maldjian, Laurienti, Kraft, & Burdette, 2003). For sensory regions/lateral pathway, these are: anterior cingulate cortex, primary somatosensory cortex and secondary somatosensory cortex. For affective regions/medial pathway, these are: amygdala, periaqueductal gray, hypothalamus, pre‐frontal cortex, insula, and hippocampus.

Standard SPM12 approaches will be used to generate first‐level T‐maps. We will model activation as the contrast of a target temperature relative to baseline or relative to warmth (including ramp up and ramp down periods as covariates of no interest in the generalized linear model). These first‐level maps containing activation changes due to the temperature contrasts (mild pain > warmth, moderate pain > warmth, and moderate pain > mild pain) will be exported from SPM for hypothesis testing (Table 1).

**TABLE 1:** Index of protocol‐relevant acronyms and their meaning

3T	3 Tesla
ASL	Arterial spin labeling
BOLD	Blood oxygenation level dependent
BPISF	Brief pain inventory‐short form
CBF	Cerebral blood flow
EPI	Echo planar imaging
fMRI	Functional magnetic resonance imaging
FOV	Field of view
FWHM	Full width half maximum
HAMD	Hamilton depression scale
IRB	Institutional review board
JNP	Just noticeable pain
*M*	Mean
MNI	Montreal neurological institute
MP	Moderate pain
MRI	Magnetic resonance imaging
pCASL	pseudo‐continuous arterial spin labeling
RF	Radio frequency
ROI	Region of interest
*SD*	Standard deviation
SENSE	Sensitivity encoding
SES	Socioeconomic status
SPM 12	Statistical parametric mapping version 12
SPSS	Statistical package for social sciences
STAI	State trait anxiety inventory
STROBE	Strengthening the reporting of observational Studies in epidemiology
TE	Echo time
TFE	Turbo field echo
TR	Repetition time
WFU	Wake forest university
WP	Weak pain

#### 2.4.4 Brain imaging covariates

Cerebral spinal fluid and motion‐related signals removed via regression (Behzadi, Restom, Liau, & Liu, 2007; Chai, Castañán, Öngür, & Whitfield‐Gabrieli, 2012), then will be low‐pass filtered at 0.1 Hz to retain the low frequencies relevant for connectivity estimation. ROI analysis will be the same as described above for thermal stimulation fMRI. Resting‐state ASL will be used as a covariate in fMRI analyses to account for baseline differences in CBF. We will use pseudocontinuous ASL (pCASL; spatial resolution = 3.44 × 3 × 44 × 7 mm^3^; TR/TI = 4000/1650 ms; slices = 17) using body coil RF transmission and 32‐channel SENSE head coil reception and inflowing blood water will be magnetically labeled using a long (1,650 ms) string of short (0.5 ms) Hanning pulses and then analyzed (Wang et al., 2002).

### 2.5 Ethical considerations

The Vanderbilt University Institutional Review Board (IRB) and ethics committee approved the study protocol. All activities included in this research protocol meet criteria for minimal risk interventions as defined by U.S. Federal Regulations and as approved by the university IRB. This research does not include vulnerable populations, as all subjects are adult volunteers with normal mental status.

Subjects will be carefully screened for psychological or medical contraindications to the proposed research methods. We will adhere to international standards and local requirements for MRI safety screening. The magnetic resonance procedure itself does not use ionizing radiation and there are no common physical risks to MRI scanning if proper procedures are followed. Some subjects develop anxiety in the MRI scanner due to the enclosed space. We will specifically and carefully inform subjects that they may end the MRI portion of the study at any time for any reason without penalty. If subjects suffer an anxiety reaction, study staff and physicians will ensure the participant is calm and safe prior to exiting the scanner area. The Medoc PATHWAYS system has built in safeguards against overheating and does not reach temperatures that could produce tissue injury. Subjects can remove the probe within seconds, as the probe is held in place by easy to grip Velcro strips and can stop the scan at any point. All spontaneous reports of adverse events by subjects, observations by clinical research staff and reports to research staff by family or healthcare providers will be investigated and reported to the university IRB as is appropriate.

Subjects will be provided with a written informed consent approved by our IRB. This consent will be reviewed in a private setting and all questions will be answered by research staff prior to obtaining participant signature. We will make every attempt to ensure that subjects are comfortable and respected. We will address any questions regarding their concerns and offer referrals to counseling or other mental health services if our screening procedures suggest such referrals are warranted. Subjects will be provided with a copy of their signed consent. A copy of the signed consent will be maintained in a secure, locked, confidential research file.

### 2.6 Data analysis

Statistical approaches for the analysis of the psychophysical and neurophysiological data will mirror each other. Age (continuous) and sex (male, female), and a term specifying the interaction of age and sex will be entered into general linear modeling modules in SPSS (psychophysical) and SPM (neurophysiological). We are hypothesizing different patterns of association of age with psycho‐ and neurophysiological data for males and females therefore the interaction term will be the primary effect of interest in these analyses. Main effects of ageing and sex will be tested; however, they will serve as covariates in the primary analyses. All data distributions will be evaluated and transformed as needed to meet the assumptions of the specific statistical methods. All statistical significance tests will maintain Type I error rates (alpha values) of no more than 0.05.

#### 2.6.1 Aim 1

The temperature at which JNP, WP, and MP is perceived (Aim 1, Hypothesis 1), and the subject's respective affective rating of the pain (Aim 1, Hypothesis 2) at each sensory level, comprise the key dependent variables for Aim 1. Descriptive and graphical summaries of the temperatures (intensities) and affective (unpleasantness) ratings will be generated at each of the three self‐reported sensory thresholds (JNP, WP, and MP), and for each of the stratified levels of age, to inform linear and possible nonlinear associations of age with the intensity and unpleasantness sensations. Linear mixed effects analysis incorporating robust variance estimation (i.e., generalized estimating equations) will be used to test the hypotheses that for a given self‐reported sensory threshold, temperatures are higher, and ratings of unpleasantness are lower with increasing age. This effect would be indicated by a statistically significant interaction of age level with self‐reported sensory level on the dependent variables (Aim 1, Hypothesis 1: temperature; Aim 1, Hypothesis 2: unpleasantness rating). A statistically significant linear or nonlinear association of age with those variables but no interaction effect will indicate an age effect on temperature and unpleasantness regardless of perceived pain.

#### 2.6.2 Aim 2

Age and sex associations with functional connectivity will be tested using the connectivity (Z) matrices for the relevant ROI for sensory and affective pain regions using the median connectivity value within the network for each subject. Linear regression analyses will be used to assess the age group effect on the overall connectivity values after co‐varying (adjusting) for regional gray matter volume and resting cerebral blood flow (CBF) as measured by pCASL generation software described below. As with the activation analyses, more specific age (years) and sex associations with activation will be assessed within each of the age groups. We will also examine the association of psychophysical data with regional brain connectivity metrics. To correct for multiple comparisons in brain imaging data we will use 3dClustSim (Cox, 1996; Cox & Hyde, 1997; Gold et al., 1998).To calculate the corrected *p* value of the brain signal in a respective ROI. This program uses the intrinsic volume and Monte Carlo simulations set at 10,000 iterations. A corrected voxel size of activation will be provided for each ROI to determine statistical significance. Data distributions will be evaluated and transformed as needed to meet the assumptions of the specific statistical methods and p‐values of 0.05 will be used for all tests.

#### 2.6.3 Missing data

This is a short protocol, with one scan day per participant; therefore, there will be no dropouts after the scan, since the scans complete the necessary procedures. We will ensure sufficient enrollment so that subjects have a full complement of usable scan data.

#### 2.6.4 Sample size estimation

Case/control groups of the size proposed in this research (30 younger adults, 30 older adults) will provide 80% statistical power to detect a difference in outcomes of interest as small as half a standard deviation in the distributions of the observed values. This represents a large difference in terms of the expected control values. Given that this is a preliminary exploratory study, we want to focus on detecting larger effects. For example, assuming that we find that the younger group reports that mild pain is experienced on average at 35°C (*SD* 6°C) with a corresponding unpleasantness rating of mean 9.2 (*SD* 4.0), our group sizes can detect an increase in the temperature level for mild pain in the older group as small as 3°C and a difference in unpleasantness ratings as small as 2 scale points. The same logic will hold for the activation and functional connectivity age group analyses. Using data from previous ageing research (Cole et al., 2006, 2010) we can expect that our control participants may have activation values (as Z scores) with approximate *mean* = 3.81 and *SD* 0.46. Given our proposed sample sizes, a respective case activation mean value of ~4.04 would be statistically significant. Correlations between observed values (e.g., pain, age, depression, anxiety, and activation in an ROI) as small in absolute value as 0.34 will be statistically significant (*p* < .05).

### 2.7 Validity and reliability/rigor

A key strength of this study is that it will be performed under routine clinical conditions using an FDA‐approved thermal pain stimulus delivery system. The single‐site nature of this study provides strong internal validity. In addition, the incorporation of SPM12 ensures that neuroimaging results adhere to long‐established methods in MRI data analysis. To ensure transparency in study conduct, the Strengthening the Reporting of Observational Studies in Epidemiology (STROBE) guidelines for cross‐sectional studies will guide the report of research results (Von Elm et al., 2007).

## 3 DISCUSSION

Pain in older adults is a widespread problem, the impact of which on public health is often underestimated, as reflected by the scarcity of research in the literature. Due to this scarcity of similar studies, this proposal is innovative in terms of both the problems that it addresses, and the interpretation of findings in the proposed hypothesized neurobiological model that permits the correlation of subjective pain reports and brain activation. Notably, thermal pain associated brain activation in certain regions is not simply correlated with pain; rather, brain activation in specific regions is a neural signature for the presence of pain in healthy individuals. This means that fMRI has enormous potential for pain assessment. The proposed methods are novel because they include a stratified sample of continuously age‐matched‐scaled younger and older adults in the same study and in the methods’ application in the context of a multimodal approach. To the best of our knowledge, this is the first study examining sex‐ and age‐associated effects on the inhibition of pain associated unpleasantness. We will also assess CBF and brain structure and examine discrepancies between sensory and affective pain responses, which enhances the relevance and interpretability of the findings. Preliminary pilot data in a sample of 12 male and 12 female (age‐matched) healthy older adults (ages 65–81) supports our proposed hypotheses and suggests that in response to thermal pain, increasing age is associated with increased brain activation and reduced unpleasantness in females only.

The procedures proposed in this protocol are similar to those used in our completed and ongoing study of thermal pain processing in dementia (Anderson et al., 2017; Cowan et al., 2017; Monroe et al., 2015, 2017, 2018; Wang, Dietrich, Simmons, Cowan, & Monroe, 2018). The principal investigators lead neuroimaging research programs focused on pain, ageing and dementia and psychiatric neuroimaging.

### 3.1 Limitations

Since recruitment, acquisition and analysis methods are routine in our labs (Monroe et al., 2015, 2017; Monroe, Gore, Gore, Chen, Mion, & Cowan, 2012) we do not expect problems in these areas. We have previously shown that cognitive status and race can have an impact on pain assessment in older adults (Monroe & Carter, 2010; Monroe, Carter, Carter, Feldt, Tolley, & Cowan, 2012). To avoid these concerns, we will recruit individuals who are cognitively intact and from matched ethnic/racial backgrounds. Socioeconomic status (SES) will be matched to the SES status of the older adult cohort because SES can also have an impact on the pain experience (Katz, 2006) We are tightly controlling for genetic variations that can have an impact on both cognition and brain activation. We will use validated interview approaches for obtaining detailed medical histories, employ systematic and rigorous multivariable statistical analytic approaches and examine results in the context of neural models grounded by basic neuroscience.

## 4 CONCLUSION

Exploring the biological reasons for alterations for sex‐differences in pain processing is essential to increasing our understanding about pain in older adults. This new knowledge will hopefully translate into information that nurses and clinicians can use to develop improved pain management recommendations tailored to the age and sex of those in their care. For instance, if hypotheses are borne out, older females may require more pain medication than younger females if the function of their endogenous opioid system is altered, whereas older males may seem less bothered by peripherally induced pain due to fewer nerve fibers and thus require less pain medication. However, for cases involving greater degrees of tissue damage, the patient's pain may rapidly increase and require greater amounts of pain medication. Each scenario requires the clinician to be highly knowledgeable of the impact of age and sex on the experience of pain. Unfortunately, the paucity of neurobiological evidence to support evidence‐based best practices of pain management in males relative to females and older adults’ places all these individuals at risk for poor pain management practice. Understanding how age and sex have an impact on central processing is a critical step to improving personalized pain medicine.

## ANONYMISED CONFLICT OF INTEREST STATEMENT

No conflicts of interest have been declared by the authors.

### PEER REVIEW

The peer review history for this article is available at https://publons.com/publon/10.1111/jan.14514.
